# Supplementary material for: Pharmacogenetic Variants Associated with Fluoxetine Pharmacokinetics from a Bioequivalence Study in Healthy Subjects
Source: J Pers Med. 2023 Sep 1;13(9):1352. doi: 10.3390/jpm13091352 (PMC10532907; doi:10.3390/jpm13091352)
Supplement: Supplementary file 1 [file jpm-13-01352-s001.zip › jpm-2552863-SI.pdf]

Supplemental Information:

Table S1. Individual fluoxetine PK parameters.

Subject	Sequence	Test product								Reference product							
		C_{max}	AUC_{0-t}	$AUC_{0-\infty}$	t_{max}	$t_{1/2}$	Vd	K_{el}	Cl	C_{max}	AUC_{0-t}	$AUC_{0-\infty}$	t_{max}	$t_{1/2}$	Vd	K_{el}	Cl
1	1	21.77	665.36	944.61	6.00	37.24	1137.47	0.02	21.17	19.24	678.45	915.85	3.00	35.89	1130.71	0.02	21.84
2	2	13.83	295.70	344.96	3.00	26.45	2212.10	0.03	57.98	10.85	274.78	316.33	3.00	24.91	2272.49	0.03	63.23
3	2	17.58	656.51	882.20	5.00	36.45	1192.09	0.02	22.67	20.99	628.97	809.15	5.00	32.38	1154.66	0.02	24.72
4	1	9.60	268.31	361.57	5.00	35.35	2820.70	0.02	55.31	7.68	230.55	329.15	6.00	44.26	3880.10	0.02	60.76
5	2	14.54	370.91	429.55	4.00	24.79	1665.08	0.03	46.56	18.03	396.11	457.89	2.00	27.19	1713.46	0.03	43.68
7	1	18.02	461.90	562.35	5.00	29.39	1507.97	0.02	35.57	16.44	389.05	449.17	5.00	25.29	1624.38	0.03	44.53
8	2	14.46	385.01	475.17	6.00	31.39	1905.91	0.02	42.09	15.68	345.59	437.44	4.00	34.23	2257.83	0.02	45.72
9	1	8.92	217.37	265.38	4.00	30.42	3307.49	0.02	75.36	8.63	210.89	255.81	6.00	30.14	3399.63	0.02	78.18
10	2	15.84	371.93	506.00	5.00	40.32	2299.01	0.02	39.53	12.85	345.33	470.59	5.00	39.05	2394.62	0.02	42.50
11	1	12.14	171.86	200.73	3.00	15.42	2216.11	0.04	99.64	7.42	157.33	171.14	4.00	19.25	3246.42	0.04	116.87
12	2	13.24	269.72	304.14	3.00	24.32	2307.71	0.03	65.76	14.23	239.53	310.17	4.00	22.37	2080.95	0.03	64.48
13	1	20.25	401.55	485.71	3.00	26.91	1598.54	0.03	41.18	17.03	440.02	538.13	3.00	30.46	1632.96	0.02	37.17
14	2	15.37	296.70	370.13	5.00	19.93	1553.58	0.03	54.04	11.81	168.42	341.88	5.00	22.76	1921.08	0.03	58.50
15	2	15.13	322.48	383.78	3.00	28.03	2107.47	0.02	52.11	15.79	301.81	335.49	2.00	23.03	1980.33	0.03	59.61
16	1	15.25	330.89	377.82	5.00	24.99	1908.07	0.03	52.93	16.05	338.47	386.55	5.00	24.58	1834.77	0.03	51.74
17	2	18.90	396.98	458.70	5.00	25.80	1623.06	0.03	43.60	16.69	353.78	403.83	5.00	24.87	1776.95	0.03	49.53
18	1	18.76	577.69	686.16	5.00	27.17	1142.58	0.03	29.15	22.77	624.48	762.94	6.00	28.19	1066.01	0.02	26.21
19	2	18.24	572.51	755.65	4.00	35.36	1350.17	0.02	26.47	23.17	583.59	699.92	3.00	28.05	1156.22	0.02	28.57
20	1	16.30	422.68	501.94	6.00	25.80	1482.84	0.03	39.85	16.15	377.00	466.07	5.00	33.07	2047.32	0.02	42.91
22	1	12.03	276.39	310.08	5.00	21.85	2032.89	0.03	64.50	13.21	304.94	345.39	5.00	22.54	1883.10	0.03	57.91
23	1	32.05	640.75	716.66	2.00	23.10	930.04	0.03	27.91	23.71	621.29	741.34	5.00	28.02	1090.50	0.02	26.98
24	2	12.80	345.82	445.73	6.00	34.77	2250.53	0.02	44.87	20.05	390.12	448.28	3.00	25.09	1614.66	0.03	44.61
25	1	24.13	957.96	2351.79	5.00	88.86	1090.16	0.01	8.50	22.73	930.62	2383.39	4.00	106.88	1293.86	0.01	8.39
26	2	10.79	202.34	224.70	3.00	23.03	2957.14	0.03	89.01	10.79	225.33	242.51	4.00	19.24	2289.59	0.04	82.47
Mean		16.25	411.64	556.06	4.42	30.71	1858.28	0.03	47.32	15.92	398.19	542.43	4.25	31.32	1947.61	0.03	49.21
Geometric mean		15.59	377.86	473.26	4.25	28.90	1761.90	0.02	42.26	15.12	360.06	457.47	4.07	29.02	1830.29	0.02	43.72

Standard deviation (S.D.)	5.02	184.05	429.46	1.18	13.78	619.84	0.01	21.32	4.89	189.55	436.65	1.19	17.21	732.91	0.01	22.98
Standard error of the mean (SEM)	1.02	37.57	87.66	0.24	2.81	126.52	0.00	4.35	1.00	38.69	89.13	0.24	3.51	149.61	0.00	4.69
Minimum	8.92	171.86	200.73	2.00	15.42	930.04	0.01	8.50	7.42	157.33	171.14	2.00	19.24	1066.01	0.01	8.39
Median	15.31	371.42	452.21	5.00	27.04	1785.49	0.03	44.24	16.10	349.68	442.86	4.50	27.60	1858.93	0.03	45.17
Maximum	32.05	957.96	2351.79	6.00	88.86	3307.49	0.04	99.64	23.71	930.62	2383.39	6.00	106.88	3880.10	0.04	116.87
Coefficient of variation (%)	30.90	44.70	77.20	26.60	44.90	33.40	28.30	45.10	30.70	47.60	80.50	28.00	55.00	37.60	26.10	46.70

Subjects IDs 6 and 21 dropped out from the bioequivalence trial.

Table S2. SNVs and demographic characteristics as predictor variables of PK parameters.

Models excluding demographic characteristics					Models including demographic characteristics				
Dependent variable	Predictor variables	Coefficient	R^2	Adj. R^2	Dependent variable	Predictor variables	Coefficient	R^2	Adj. R^2
$AUC_{0\text{-}t}$ (h*ng/mL)	Constant	-384.909	0.984	0.977	$AUC_{0\text{-}t}$ (h*ng/mL)	Constant	1407.983	0.984	0.978
	ABCB1(rs1128503)	-6.563				*ABCB1*(rs1045642)	-35.575		
	CYP1A2(rs2470890)	-33.751				*ABCB1*(rs1128503)	-5.932		
	CYP1A2(rs762551)	-18.890				*CYP1A2*(rs2069514)	-36.506		
	CYP2C19(rs4244285)	12.884				*CYP1A2*(rs2470890)	-22.919		
	CYP2C9(rs1799853)	26.770				*CYP2B6*(rs2279342)	-64.374		
	CYP2C9(rs28371686)	74.827				*CYP2C19*(rs4917623)	29.571		
	CYP2D6(rs1065852)	-55.760				*CYP2C9*(rs1799853)	-12.555		
	CYP2D6(rs1135840)	23.740				*CYP2D6*(rs1135840)	15.952		
	CYP2D6(rs28371703)	137.454				*CYP2D6*(rs28371703)	78.419		
	CYP2D6(rs28371706)	-118.191				*CYP3A4*(rs2687116)	-7.826		
	CYP2D6(rs72549358)	115.010				*CYP3A5*(rs776746)	-53.635		
	CYP3A4(rs2740574)	-126.951				Gender	-131.696		
	CYP3A4(rs3735451)	-29.337				Height (m)	-257.222		
	SLC6A4 (rs1042173)	13.325				*TPH1* (rs1799913)	-20.761		
	SLC6A4 (rs2066713)	16.080							
Cl (L/h)	Constant	567.486	0.980	0.969	Cl (L/h)	Constant	103.181	0.955	0.940
	ABCB1(rs2032582)	-1.280				*CYP1A2*(rs2470890)	3.452		
	CYP1A2(rs2470890)	5.436				*CYP2C19*(rs11188072)	3.238		
	CYP2B6(rs2279344)	-5.530				*CYP2C9*(rs28371686)	-3.142		
	CYP2B6(rs4803418)	1.582				*CYP2D6*(rs1065852)	6.809		
	CYP2C19(rs11188072)	2.272				*CYP2D6*(rs16947)	-6.315		
	CYP2C19(rs12769205)	5.584				*CYP2D6*(rs28371703)	-19.606		
	CYP2C19(rs4917623)	1.555				*CYP2D6*(rs28371706)	19.316		
	CYP2C9(rs1799853)	-4.696				*CYP3A4*(rs2242480)	-3.734		
	CYP2C9(rs2256871)	4.406				*CYP3A5*(rs776746)	6.012		

	CYP2C9(rs28371686)	-9.384				Age (Years)	-0.520		
	CYP2D6(rs16947)	-3.932				Gender	20.301		
	CYP2D6(rs28371703)	-19.079				*SLC6A4* (rs1042173)	-2.145		
	CYP2D6(rs28371706)	26.620							
	CYP3A4(rs67666821)	-45.090							
	SLC6A4 (rs1042173)	-5.815							
	SLC6A4 (rs2066713)	-1.428							
$t_{1/2}$ (h)	Constant	-1.100	0.935	0.924					
	CYP2B6(rs35303484)	3.229							
	CYP2C9(rs2256871)	10.096							
	CYP2D6(rs1065852)	-13.304							
	CYP2D6(rs28371703)	15.256							
	CYP2D6(rs28371706)	-13.635							
	CYP3A4(rs2740574)	9.739							
	TPH1 (rs1799913)	-0.696							

Only significant models are shown ($p < 0.000$) with significant predictor variables ($p < 0.05$)
